# Patient‐Related Factors Influencing Motivation to Change in Adolescents With First‐Onset Anorexia Nervosa: A Cross‐Sectional Study

**DOI:** 10.1002/erv.3182

**Published:** 2025-02-15

**Authors:** Elisabeth De Mey, Marisha N. Meijer, Katrien F. M. Bracké, Cathelijne P. M. Steegers, Manon H. J. Hillegers, Marina Danckaerts, Tonya White, Gwendolyn C. Dieleman

**Affiliations:** ^1^ Department of Child and Adolescent Psychiatry University Hospitals Leuven Leuven Belgium; ^2^ Department of Child and Adolescent Psychiatry/Psychology Erasmus MC Sophia Children's Hospital Rotterdam the Netherlands; ^3^ Department of Radiology and Nuclear Medicine Erasmus MC Rotterdam The Netherlands; ^4^ Section on Social and Cognitive Developmental Neuroscience National Institutes of Mental Health Bethesda Maryland USA

**Keywords:** adolescent, anorexia nervosa, first onset, motivation to change, RMQ

## Abstract

**Objective:**

Motivation to change significantly impacts treatment outcomes in eating disorders (EDs). This study investigated patient‐related factors associated with motivation to change in adolescents with first‐onset anorexia nervosa (AN). Understanding these factors will help tailor interventions to individual needs, enhancing treatment outcomes.

**Method:**

Seventy‐six female adolescents with first‐onset AN completed the Readiness and Motivation Questionnaire (RMQ)—Dutch translation. ED symptoms, body mass index, and comorbidity (depressive, anxiety, obsessive‐compulsive, autism spectrum disorder, and attention deficit and hyperactivity disorder symptoms) were analysed using linear regression analyses. As a secondary aim, the association between specific ED behaviours and motivation to change was analysed. This project was preregistered: https://osf.io/vx9ud/.

**Results:**

Adolescents with more severe ED symptoms, depressive symptoms, and obsessive‐compulsive symptoms, experienced lower motivation to change. The severity of ED symptoms emerged as the most important factor associated with motivation to change, specifically ED symptoms concerning weight and body shape. Additionally, laxative misuse was associated with greater motivation to change.

**Conclusions:**

The link between motivation to change and ED symptoms suggests that early treatment can improve outcomes by reducing ED symptoms and facilitating change. Furthermore, addressing depression during treatment might enhance motivation to change.

**Summary**

- This study aimed to better understand which factors are associated with motivation to change in adolescents with first‐onset anorexia nervosa (AN) using a Dutch translation of the Readiness and Motivation Questionnaire (RMQ).
- Having more severe ED symptoms, specifically concerns about shape and weight, is associated with lower motivation to change. The effect that comorbid symptoms (depressive, obsessive‐compulsive symptoms) have on motivation to change is in part explained by the severity of ED symptoms.
- We found that the RMQ was able to detect variation in motivation to change in an adolescent first‐onset AN sample, which supports the possible application of this instrument in Dutch clinical care.

## 1 Introduction and Aims

Anorexia nervosa (AN) is a severe and debilitating psychiatric illness, characterised by high rates of relapse and chronicity, making it challenging to treat (Kaplan et al. 1999). Long‐term outcomes highlight the gravity of this condition; a longitudinal study reported that only 30% of individuals with AN achieved remission after 10‐year (Fichter et al. 2017). Among the many factors influencing treatment and recovery, motivation to change has emerged as an important component in understanding and improving therapeutic outcomes (Vall and Wade 2015).

Given its important role, motivational techniques are a key element of all AN treatment programs, and treatment guidelines (GGZ Standaarden 2017; Practice Guidelines 2023) emphasise the necessity of patients being motivated to change their eating disorder (ED) behaviours. Supporting this, systematic reviews (Gregertsen et al. 2019; Sansfaçon et al. 2020) and multiple clinical studies (Schlegl et al. 2014; Hillen et al. 2015; McHugh 2007; Castro‐Fornieles et al. 2007; Ametller et al. 2005) have identified motivation to change as a positive predictive factor for AN recovery. In these studies, it was found that patients who were more motivated to change their ED exhibited greater improvement in ED symptoms (Schlegl et al. 2014; Hillen et al. 2015), more weight gain (Hillen et al. 2015), shorter lengths of hospitalisation (McHugh 2007), better weight maintenance after discharge (Castro‐Fornieles et al. 2007), and a smaller chance of hospital admission at follow‐up (Ametller et al. 2005). Therefore, it is important to gain an understanding of which factors can influence patients' motivation to change.

In research, there are several models that conceptualise behaviour change. Most of them are based on the Transtheoretical model of behaviour change by Prochaska and Diclemente (TTM; Prochaska, DiClemente, and Norcross 1992), which is a frequently used theoretical framework to describe motivation in EDs. The TTM describes six stages of readiness for change (precontemplation, contemplation, preparation, action, maintenance, termination), with ascending stages indicating an increase in readiness to change. Other aspects of motivation are the degree of internal versus external motivation to change (Ryan et al. 2000), and having confidence in the ability to change, which is described by Bandura (Bandura 1997) as self‐efficacy.

A growing number of studies have investigated factors that can influence motivation to change in EDs. So far, several variables have been identified as being associated with motivation to change in EDs: patient‐related variables (such as demographic, disease‐related, comorbidity‐related, and psychological variables) and context‐related variables (such as adaptivity of the parent‐child relationship, friendships, relationships with healthcare professionals). However, most of these studies used heterogeneous ED samples, combining participants with AN, BN, and atypical EDs. Considering that it has been demonstrated that motivation to change differs between various EDs (Ackard et al. 2015), results from heterogeneous ED samples are possibly not applicable to AN‐only samples.

When focusing on AN‐only samples, most research has been conducted with patient‐related variables (specifically, demographic, disease‐, and comorbidity‐related variables). A negative association between motivation to change and the severity of ED symptoms was established in the largest number of studies (Hillen et al. 2015; McHugh 2007; Green et al. 2017; Serrano et al. 2004). Also, a negative association with depressive symptoms (McHugh 2007; Serrano et al. 2004), and anxiety symptoms (McHugh 2007) was found. However, one study found no significant association between motivation to change and depressive symptoms (Hillen et al. 2015). For other previously investigated variables such as BMI, age, and duration of illness, the literature remains unclear. Regarding BMI, it is generally hypothesised that low BMI contributes to low motivation to change in patients with AN through the effects of starvation on reward and control processes in the brain (Treasure et al. 2011). Yet, clinical studies have found no correlation between BMI and motivation to change (Geller et al. 2013), or even more surprisingly, have found that patients with lower BMI showed more motivation to change (Hillen et al. 2015; McHugh 2007). Regarding age‐related differences in motivation to change in AN samples, research is scarce. Two studies found no association between age and motivation to change (McHugh 2007; Hasler et al. 2004), although Hillen (Hillen et al. 2015) observed that younger AN patients perceived greater coercion, which could be indicative of a lack of internal motivation to change. Considering the effect of duration of illness on motivation to change, results are mixed; one study (Hillen et al. 2015) observed a positive association between illness duration and motivation to change, and another found no association (Hasler et al. 2004). In other words, there is considerable uncertainty about the potential influence that BMI, age, and illness duration, have on motivation to change in patients with AN. Furthermore, most studies examining motivation to change in AN included adults; these results might not be applicable to adolescents with a relatively shorter illness duration.

Although some of the differences in results between previous studies could be explained by differences in sample characteristics such as ED type, age, duration of illness, and treatment setting, this does not account for all differences. This emphasises that the underlying motivation dynamics in AN are still not fully understood and possibly other, yet unknown, factors are at play. For instance, there are other patient‐related variables that may influence patients' motivation to change that have been identified in previous research as having an influence on ED treatment outcome, but their relation to motivation to change has not been studied, or outcomes have not been reported. For example, more autism spectrum disorder (ASD) symptoms have been linked to less change in ED symptoms after 1 year of treatment (Stewart et al. 2017), and to longer AN illness duration (Saure et al. 2020). More attention‐deficit/hyperactivity disorder (ADHD) symptoms have been associated with the persistence of ED diagnosis after 1 year of treatment (Svedlund et al. 2018), and obsessive‐compulsive traits have been linked to worse outcome at 18‐year follow‐up (Wentz et al. 2009). In addition, most studies examined singular associations between predictor variables and motivation to change, while a model in which several predictors are entered simultaneously, might give more insight into the most relevant predictors that drive motivation to change.

In conclusion, we have identified three gaps in the literature, which will be addressed in the current study by including a homogeneous adolescent AN sample, examining variables previously investigated in motivation research as well as those not yet explored, and analysing the combined effect of these variables. Through this approach, we aim to develop a more nuanced understanding of the patient‐related factors influencing motivation to change in patients with AN. To the best of our knowledge, this is the first study to investigate motivation to change in a female adolescent first‐onset AN sample, and the first study to examine the combined effect of a multitude of patient‐related variables on motivation to change. Furthermore, this study provides initial support for the use of the Readiness and Motivation Questionnaire (RMQ) in Dutch clinical care.

## 2 Methods

### 2.1 Aims and Study Designs

This study was part of the larger research project BRAVE (Steegers et al. 2024), a longitudinal multicenter research project initiated within the Erasmus University Medical Centre, Sophia Children's hospital in Rotterdam, the Netherlands. The BRAVE project investigates biological, cognitive, and psychological factors in adolescents with first‐onset AN with the aim of gaining better insight in the underlying mechanisms of AN. The BRAVE project was conducted between May 2017 and January 2023. In this study, we used baseline data in a cross‐sectional design. The research protocol was approved by the medical ethics committee of the Erasmus Medical Center, Rotterdam (MEC 2016‐194/NL55175.078.16). This project was preregistered: https://osf.io/vx9ud/.

### 2.2 Participants and Data Collection

The total sample consists of 79 female participants aged 12–22 years, with a DSM‐5 classification of first‐onset typical or atypical AN, classified in the past 12 months, partaking in treatment at one of the participating clinics. Participants were recruited from 16 mental health institutions and hospitals in the Netherlands, and via advertisements on social media and through patient organisations. After obtaining informed consent, questionnaires and interviews assessing motivation, ED symptoms, and comorbid psychopathology were administered in Dutch, and physical measurements were taken. Readiness and Motivation Questionnaire (RMQ) scores were available for 76 out of the 79 participants; data from these 76 participants will be evaluated. More detailed information about the data collection procedure, and inclusion and exclusion criteria, is available in the study design paper (Steegers et al. 2024).

### 2.3 Measures

#### 2.3.1 Motivation to Change

We measured motivation to change using a Dutch translation of the Readiness and Motivation Questionnaire (RMQ (Geller et al. 2013);), shared with permission by the developer and shown in Supporting Information S1: Appendix 1), which was translated in collaboration with psychologists, psychiatrists and a professional translation service (Metamorfose Vertalingen BV, official translating partner of Erasmus University Rotterdam, 2021 (shown in Supporting Information S1: Appendix 2) for the purpose of this study. The Dutch translation is available from the authors. The RMQ is a 60‐item questionnaire derived from the Readiness and Motivation Interview (RMI; Geller et al. 1999), and based on the Transtheoretical model of change (Prochaska, DiClemente, and Norcross 1992). The RMQ provides two ‘stages of change’ scores that represent the degree of readiness to make behaviour changes, that is precontemplation (not intending to take action in the foreseeable future [Prochaska, DiClemente, and Norcross 1992]), and action (specific, overt modifications in behaviour have been made in the recent past [Prochaska, DiClemente, and Norcross 1992]). It also provides two additional motivational domains, that is internality and confidence, reporting respectively the degree of changing for internal versus for external reasons, and the confidence someone has in the ability to change.

As previous research has indicated that variables can be related to one, but not necessarily all RMQ scales (Iyar et al. 2019), we chose to include all four RMQ total scores (precontemplation, action, internality, and confidence) in this study. RMQ total scores are continuous and range from 0 to 100. Lower readiness to change is indicated by higher RMQ precontemplation total scores and lower RMQ action total scores. Individuals who change for internal reasons are indicated by higher RMQ internality total scores, while those who have more confidence in their ability to change their behaviour are indicated by higher RMQ confidence total scores.

The RMQ has shown to be a valid instrument with fair test‐retest reliability and good convergent and discriminant validity (Geller et al. 2013) in a mixed ED sample.

#### 2.3.2 Eating Disorder Pathology

The Eating Disorder Examination (EDE 12.0; Fairburn et al. 1993; Jansen 2000) is a semi‐structured interview that is considered the gold standard (e.g., Guest 2000) for measuring ED symptoms and their severity. It consists of 34 items, 23 of which are used to generate the following four subscales: restraint, eating concern, shape concern, and weight concern. An EDE total score is also obtained. Higher EDE total and subscale scores indicate more severe ED pathology. The EDE subscales demonstrate low to high internal consistency (*α* = 0.51–0.85) in clinical samples (Berg et al. 2011).

Since previous research showed that motivation to change can differ between the different ED symptom types (Kambanis et al. 2022), not only the EDE total score but also the four EDE subscale scores were used in our primary analysis. Results on EDE questions investigating the presence of binge‐eating, self‐induced vomiting, and laxative misuse (EDE questions nr. 9, 14 and 15, respectively), were used separately to investigate a possible effect of these behaviours on motivation to change.

#### 2.3.3 Comorbid Psychopathology

The Beck Depression Inventory (BDI‐II, NL; Wang et al. 2013; Van der Does 2002), is a self‐report questionnaire that measures depressive symptoms. It has 21 items that are scored on a 4‐point Likert scale. A BDI total score is obtained, with higher scores indicating the presence of more depressive symptoms. The BDI has a high internal consistency (BDI‐II‐NL [Van der Does 2002] *α* = 0.88–0.92) and a good convergent validity (Van der Does 2002).

The Screen for Child Anxiety Related Disorders (SCARED‐NL; Muris et al. 1998; Muris et al. 2007), is a self‐report questionnaire for anxiety symptoms. It consists of 69 items that are answered on a 3‐point Likert scale. A total score is obtained, with higher scores indicating the presence of more anxiety symptoms. The SCARED has high internal consistency (SCARED [Hale et al. 2005] *α* = 0.93).

The (Children's) Yale‐Brown Obsessive Compulsive Scale ((C)Y‐BOCS; [Scahill et al. 1997; de Haan et al. 2007; Goodman et al. 1989]), is a semi‐structured interview that assesses obsessive and compulsive behaviours and thoughts. CY‐BOCS questionnaires are administered to participants aged 12–17 years; Y‐BOCS questionnaires to participants aged 18–22 years. Both versions of the interview consist of 10 items that are scored on a 5‐point scale. For each participant, the total score of the appropriate questionnaire is used; higher total scores indicate the presence of more obsessive‐compulsive traits. The Y‐BOCS and CY‐BOCS have high internal consistency (Y‐BOCS [Woody, Steketee, and Chambless 1995] *α* = 0.88–0.91; CY‐BOCS [Scahill et al. 1997] *α* = 0.87) and good convergent validity (Scahill et al. 1997; Woody, Steketee, and Chambless 1995).

The Social Responsiveness Scale (Adult) (SRS(‐A) (Constantino et al. 2003, 2012; Roeyers et al. 2011; Noens, De la Marche, and Scholte 2012) is a parent‐reported questionnaire for Autism Spectrum Disorder (ASD) symptoms. For participants aged 12–17 years, the SRS‐2 will be completed, and for participants aged 18–22 years the SRS‐A will be completed. The SRS‐2 consists of 65 questions, and the SRS‐A of 64 questions. Both questionnaires are answered on a 4‐point Likert scale. Total scores are calculated and converted to t‐scores, with higher scores indicating the presence of more ASD symptoms. For each participant, the appropriate SRS t‐score will be used. The SRS‐2 and the SRS‐A have a high internal consistency (SRS‐2 [Roeyers et al. 2011] *α* = 0.93; SRS‐A [Chan et al. 2017] *α* = 0.90) and good convergent validity (Roeyers et al. 2011; Chan et al. 2017).

The Child/Adult Behaviour Checklist (CBCL/ABCL; Verhulst, Van der Ende, and Koot 1996; Achenbach et al. 2003), is a questionnaire used to assess a wide range of behavioural and emotional problems. We used the CBCL for participants aged 12–17 years (parent report), and the ABCL for participants aged 18–22 years (parent report). The questionnaires contain a number of items that are answered on a 3‐point Likert scale; higher scores indicating the presence of more symptoms. For both questionnaires, total scores of the DSM‐scale ADHD are used, with higher scores indicating more ADHD symptoms. The DSM‐scale ADHD has a high internal consistency (CBCL [Verhulst, Van der Ende, and Koot 1996] *α* = 0.82–0.89; ABCL [Achenbach et al. 2003] *α* = 0.83).

The Mini‐International Neuropsychiatric Interview (MINI‐KID/MINI‐PLUS (Sheehan et al. 2010; Bauhuis et al. 2013; Sheehan et al. 1998; van Vliet et al. 2000);) assesses general psychopathology and is a widely used structured diagnostic interview. The MINI‐KID (< 17 years) consists of 23 modules and the MINI‐PLUS (> 18 years) consists of 26 modules, with each module corresponding to a diagnostic category. All modules start with one or more screening questions that determine the possibility of a diagnosis. Both versions of the MINI yield DSM‐IV diagnoses (the DSM‐5 update was not yet available at the start of inclusion). The MINI‐KID/MINI‐PLUS have good to excellent sensitivity and specificity (Sheehan et al. 1998, 2010).

#### 2.3.4 Standardised Body Mass Index

The standardised body mass index (BMI‐SDS) is calculated for each participant by adjusting BMI scores (kg/(m^2^)) for age and sex. Growth curves from the Dutch Organisation for Applied Research (TNO) were used to calculate BMI‐SDS (Cole et al. 2007).

### 2.4 Statistical Analyses

Descriptive statistics were used to calculate response rates, mean values, and to visualise data distribution.

The internal consistency of the Dutch translation of the RMQ in our adolescent first‐onset AN sample was investigated by calculating Cronbach's alpha coefficients for the precontemplation, action, internality, and confidence total scores. Question 8 (pertaining to the use of diuretics) was omitted from analysis as this item was non‐applicable for all participants.

To identify predictor variables that are associated with motivation to change, we first conducted a series of simple linear regression analyses, entering the four RMQ total scores (precontemplation, action, internality, and confidence) as *dependent* variables. The following variables were entered as *independent* variables: EDE total scores and EDE subscales restraint, eating concern, shape concern, weight concern scores, BDI total score, SCARED‐NL total score, BMI‐SDS, (C)Y‐BOCS total scores, SRS(‐A) t‐scores, and CBCL/ABCL ADHD scale scores. Based on previous research, we hypothesised that motivational scores would be negatively associated with EDE, BDI, and SCARED scores. The other variables were included in exploratory analyses. To account for possible multiple testing effects, we corrected significance levels using the Benjamini‐Hochberg formula for False Discovery Rate (FDR).

To construct a predictive model that explains how these variables are associated with patients' motivation to change, the relevant predictors from the simple linear regression analyses were entered into a multiple linear regression analysis. This analysis was conducted twice: once including and once excluding the EDE total score. This approach was chosen based on the literature (Hillen et al. 2015; McHugh 2007; Green et al. 2017; Serrano et al. 2004), which has already established a strong association between ED symptomology and motivation to change. Therefore, excluding ED symptomology will provide a more comprehensive understanding of the specific association between the comorbid symptoms and motivation to change.

We considered age to be a possible covariate and corrected all analyses for age. Since our sample consists of a homogeneous sample with a mean illness duration of 5 months, we decided not to use illness duration as a separate independent variable or covariate.

As a secondary aim, we aimed to determine whether specific ED behaviours, including binge‐eating, self‐induced vomiting, and laxative misuse, were associated with motivation to change. To this end, we employed independent sample *T*‐tests to compare the mean RMQ total scores for the group of participants who exhibited these behaviours with the group of participants who did not. Subsequently, for significant group differences, we conducted a one‐way between‐groups analysis of covariance (ANCOVA) to take into account possible confounding effects of age and severity of ED symptoms.

#### 2.4.1 Non‐Response Analysis

For variables with more than 10% missing data, non‐response analyses were performed comparing participants *with* versus *without* missing data (see Table 1). A notable clustering of missing data was observed on the self‐report questionnaires, with BDI and SCARED scores missing for the same participants. A similar pattern was observed on the parent‐report questionnaires, with SRS(‐A) and CBCL/ABCL ADHD scores missing for the same participants. BDI and SCARED data were more frequently missing in participants with higher EDE restraint scores (*p* < 0.05), higher EDE total scores (*p* < 0.05), younger participants (*p* < 0.05), and participants with higher CBCL/ABCL ADHD scores (*p* < 0.01). SRS(‐A) and CBCL/ABCL ADHD data were more frequently missing in participants with higher EDE weight concern scores (*p* < 0.05) and in older participants (*p* < 0.05). Participants did not differ in their scores for any of the RMQ scales, or for BMI‐SDS.

## 3 Results

### 3.1 Sample Characteristics

Table 1 presents the baseline characteristics and the RMQ total scores. Our sample had a mean age of 16 years and predominantly consisted of participants with typical AN. The vast majority of participants received outpatient treatment at time of inclusion. A structured assessment of comorbidity showed high rates of comorbid mood and anxiety disorders. RMQ precontemplation scores were relatively high compared to RMQ action, internality and confidence scores, which indicates low levels of motivation to change.

**TABLE 1:** Baseline characteristics and the RMQ total scores.

	*N*	Mean (SD)/%
Age (years)	76	16.58 (2.22)
BMI‐SDS	76	−1.43 (1.31)
Illness duration (months)	69	5.00 (3.56)
*AN diagnosis*	76	
Typical	72	94.7%
Atypical	4	5.3%
*Treatment*	76	
Outpatient	74	97.4%
Inpatient	2	2.6%
*Pre‐menarche*	76	
No	66	86.8%
Yes	10	13.2%
*Ethnicity*	72	
Dutch	66	91.7%
(Other) Western	5	6.9%
Non‐Western	1	1.4%
*Education level mother*	70	
Low	0	0.0%
Middle	35	50.0%
High	35	50.0%
*DSM‐IV comorbid disorders*	76	
Any mood disorder	47	61.8%
Any anxiety disorder	33	43.4%
Any OCD	16	21.1%
Any behaviour disorder	3	3.9%
Any ADHD	3	3.9%
Any ASD	0	0%
*Motivation to change*		
RMQ precontemplation	76	62.60 (21.93)
RMQ action	76	31.98 (18.20)
RMQ internality	76	42.10 (25.45)
RMQ confidence	76	26.00 (16.1Z)
*Comorbid symptoms*		
BDI	63	27.54^a^ (12.34)
SCARED	62	44.29^b^ (22.55)
(C)Y‐BOCS	72	10.44^c^ (10.60)
SRS(‐A)	64	57.02^d^ (10.31)
CBCL/ABCL ADHD	64	3.59^e^ (3.24)
*Eating disorder symptoms*		
EDE total	76	3.50 (1.20)
EDE shape concern	76	4.24 (1.43)
EDE restraint	76	3.17 (1.48)
EDE eating concern	76	2.72 (1.08)
EDE weight concern	76	3.90 (1.55)
EDE Q.9 binge eating	75	12.83 (31.35)
Present	25	
EDE Q.14 self‐induced vomiting	76	1.22 (4.63)
Present	10	
EDE Q.15 laxative misuse	76	0.97 (4.29)
Present	8	

*Note:* Ethnicity is based on country of origin of the participant's mother and categorised based on the definition by Statistics Netherlands (CBS [Statistics Netherlands]). Education level mother was divided into the categories Low (primary education), Middle (general secondary education and secondary vocational education), and High (higher vocational education and academic education). Illness duration is the interval between the date of diagnosis and the date of study inclusion expressed in months. Treatment is defined as in‐ or outpatient treatment at time of inclusion. DSM‐IV Comorbid disorders encompass DSM‐IV MINI‐KID/MINI PLUS classifications. EDE Q.9 is question 9 from the EDE (the number of subjective binge‐eating episodes occurring in the previous month). EDE Q.14 is question 14 from the EDE (the number of self‐induced vomiting episodes occurring in the previous month). EDE Q.15 is question 15 from the EDE (the number of times there was laxative misuse in the previous month). CBCL/ABCL ADHD is the ADHD DSM‐scale from the CBCL/ABCL questionnaire.

Abbreviations: ADHD, attention‐deficit/hyperactivity disorder; AN, anorexia nervosa; ASD, autism spectrum disorder; BDI, Beck Depression Inventory; BMI‐SDS, Body Mass Index Standard Deviation Score; CBCL/ABCL, Child/Adult Behaviour Checklist; (C)Y‐BOCS, (Child)‐Yale Brown Obsessive Compulsive Scale; ED, eating disorder; EDE, Eating Disorder Examination; OCD, obsessive compulsive disorder; RMQ, Readiness and Motivation Questionnaire; SCARED, Screen for Child Anxiety Related Disorders; SRS(‐A), Social Responsiveness Scale (Adult).

^a^ A mean BDI score of 27.54 corresponds to ‘moderate’ depressive symptoms.

^b^ A mean SCARED score of 44.29 ‘may indicate the presence of an anxiety disorder’.

^c^ A mean (C)Y‐BOCS score of 10.44 corresponds to ‘mild’ obsessive‐compulsive symptoms.

^d^ A mean SRS(‐A) t‐score of 57.02 corresponds to ‘within normal limits’ reciprocal social behaviour.

^e^ A mean CBCL/ABCL ADHD score of 3.59 corresponds to within ‘normal range’ attention deficit/hyperactivity problems.

### 3.2 Internal Consistency RMQ

In our sample the RMQ had moderate internal consistency: Precontemplation *α* = 0.71, Action *α* = 0.64, Internality *α* = 0.73, Confidence *α* = 0.63. There were some negative inter‐item correlations; these results can be found in Supporting Information S1: Appendix 3: RMQ validity and reliability. The coefficients from the RMQ development paper (Geller et al. 2013) are, respectively: *α* = 0.55, *α* = 0.66, *α* = 0.80, and *α* = 0.77.

### 3.3 The Association of ED Psychopathology, BMI‐SDS, and Comorbid Psychopathology With Motivation to Change

For descriptive purposes, the correlations between the independent variables are presented in Table 2. The BDI scores were found to have a strong relationship with EDE total, EDE shape concern, EDE food concern, and SCARED scores. Table 3A and 3B present the associations between the predictors and RMQ scale scores. Higher scores on the EDE, BDI, and (C)Y‐BOCS, were significantly associated with higher RMQ precontemplation scores, indicating lower readiness to change. Additionally, higher EDE and BDI scores were associated with lower RMQ internality scores, indicating lower levels of internal motivation to change; higher EDE scores were associated with lower RMQ confidence scores, indicating lower levels of confidence in the ability to change. On a subscale level, the EDE domains shape concern and weight concern had the strongest association with RMQ precontemplation and internality scores; EDE shape concern was the only EDE score that was significantly associated with RMQ confidence scores. None of the predictors demonstrated a significant association with the RMQ action scale.

**TABLE 2:** Correlations between the independent variables.

	Age	EDE total	EDE shape concern	EDE restraint	EDE eating concern	EDE weight concern	BMI‐SDS	BDI	SCARED	(C)Y‐BOCS	SRS(‐A)	CBCL/ABCL ADHD
Age	1											
EDE total	0.06	1										
EDE shape concern	0.05	0.94**	1									
EDE restraint	−0.07	0.81**	0.67**	1								
EDE eating concern	0.08	0.83**	0.73**	0.59**	1							
EDE weight concern	0.16	0.88**	0.85**	0.53**	0.64**	1						
BMI‐SDS	−0.05	−0.09	−0.06	−0.07	−0.10	−0.07	1					
BDI	0.22	0.54**	0.58**	0.32*	0.53**	0.48**	−0.18	1				
SCARED	0.12	0.31*	0.36**	0.08	0.40**	0.27*	−0.02	0.55**	1			
(C)Y‐BOCS	0.21	0.25*	0.21	0.02	0.41**	0.26*	−0.25*	0.36**	0.45**	1		
SRS(‐A)	0.39**	0.15	0.16	0.12	0.15	0.09	−0.11	0.17	0.40**	0.21	1	
CBCL/ABCL ADHD	0.23	0.17	0.20	0.13	0.09	0.15	−0.03	−0.14	0.11	0.01	0.48**	1

*Note:* Pearson correlation coefficients are represented. CBCL/ABCL ADHD is the ADHD DSM‐scale from the CBCL/ABCL questionnaire.

Abbreviations: ADHD, attention‐deficit/hyperactivity disorder; BDI, Beck Depression Inventory; BMI‐SDS, Body Mass Index Standard Deviation Score; CBCL/ABCL, Child/Adult Behaviour Checklist; (C)Y‐BOCS, (Child)‐Yale Brown Obsessive Compulsive Scale; EDE, Eating Disorder Examination; SCARED, Screen for Child Anxiety Related Disorders; SRS(‐A), Social Responsiveness Scale (Adult).

**p < 0.01, *p < 0.05 (2‐tailed).

**TABLE 3A:** Predictors of motivation to change for RMQ precontemplation and RMQ action scales.

	RMQ precontemplation					RMQ action				
	Beta	*B*	95% CI	*R* ^2^ change	*p*	Beta	*B*	95% CI	*R* ^2^ change	*p*
EDE total	0.72	13.13	10.15, 16.11	0.51**	< 0.001	−0.21	−3.21	−6.68, 0.27	0.04	0.070
EDE shape concern	0.68	10.44	7.83, 13.05	0.46**	< 0.001	−0.21	−2.60	−5.50, 0.30	0.04	0.079
EDE restraint	0.56	8.35	5.49, 11.22	0.32**	< 0.001	−0.18	−2.18	−5.01, 0.66	0.03	0.130
EDE eating concern	0.59	12.05	8.22, 15.88	0.35**	< 0.001	−0.16	−2.70	−6.60, 1.20	0.03	0.171
EDE weight concern	0.66	9.41	6.89, 11.92	0.43**	< 0.001	−0.20	−2.29	−5.02, 0.43	0.04	0.098
BMI‐SDS	−0.21	−3.57	−7.37, 0.23	0.05	0.065	0.11	1.48	−1.74, 4.69	0.01	0.364
BDI	0.53	0.93	0.53, 1.34	0.26**	< 0.001	−0.16	−0.24	−0.63, 0.14	0.03	0.215
SCARED	0.26	0.25	0.00, 0.49	0.07*	0.048	−0.11	−0.09	−0.30, 0.12	0.01	0.387
(C)Y‐BOCS	0.31	0.64	0.16, 1.12	0.09**	0.010	−0.14	−0.25	−0.66, 0.17	0.02	0.240
SRS(‐A)	0.18	0.38	−0.20, 0.96	0.03	0.198	−0.17	−0.29	−0.78, 0.19	0.02	0.235
CBCL/ABCL ADHD	−0.04	−0.27	−2.04, 1.50	0.00	0.762	−0.01	−0.03	−1.50, 1.44	0.00	0.969

*statistically significant association before application of False discovery rate correction (p < 0.05).

**statistically significant association after application of False discovery rate correction (q* < 0.0159); q* = corrected significance level by applying Benjamini‐Hochberg formula for False discovery rate.

**TABLE 3B:** Predictors of motivation to change for RMQ internality and RMQ confidence scales.

	RMQ internality					RMQ confidence				
	Beta	B	95% CI	*R* ^2^ change	*p*	Beta	B	95% CI	*R* ^2^ change	*p*
EDE total	−0.43	−9.05	−13.55, −4.55	0.18**	< 0.001	−0.26	−3.53	−6.55, −0.52	0.07*	0.022
EDE shape concern	−0.41	−7.25	−11.04, −3.46	0.17**	< 0.001	−0.28	−3.16	−5.66, −0.65	0.08**	0.014
EDE restraint	−0.37	−6.39	−10.13, −2.65	0.14**	0.001	−0.18	−2.01	−4.50, 0.48	0.03	0.113
EDE eating concern	−0.31	−7.25	−12.51, −1.99	0.09**	0.008	−0.23	−3.40	−6.78, −0.01	0.05*	0.049
EDE weight concern	−0.41	−6.80	−10.35, −3.25	0.17**	< 0.001	−0.21	−2.21	−4.60, 0.18	0.04	0.069
BMI‐SDS	0.10	1.89	−2.62, 6.39	0.01	0.406	0.14	1.68	−1.14, 4.50	0.02	0.239
BDI	−0.38	−0.78	−1.29, −0.27	0.14**	0.003	−0.15	−0.20	−0.54, 0.14	0.02	0.251
SCARED	−0.16	−0.19	−0.48, 0.11	0.03	0.211	−0.06	−0.04	−0.23, 0.15	0.00	0.668
(C)Y‐BOCS	−0.14	−0.32	−0.91, 0.26	0.02	0.272	−0.19	−0.29	−0.65, 0.08	0.03	0.121
SRS(‐A)	−0.27	−0.68	−1.34, −0.01	0.06*	0.046	−0.19	−0.30	−0.73, 0.12	0.03	0.159
CBCL/ABCL ADHD	−0.08	−0.59	−2.65, 1.46	0.01	0.566	−0.16	−0.81	−2.09, 0.47	0.03	0.209

*Note: R* ^2 change^ is based on original complete cases. All analyses were corrected for age at baseline. CBCL/ABCL ADHD is the ADHD DSM‐scale from the CBCL/ABCL questionnaire.

Abbreviations: ADHD, attention‐deficit/hyperactivity disorder; BDI, Beck Depression Inventory; BMI‐SDS, Body Mass Index Standard Deviation Score; CBCL/ABCL, Child/Adult Behaviour Checklist; (C)Y‐BOCS, (Child)‐Yale Brown Obsessive Compulsive Scale; EDE, Eating Disorder Examination; RMQ, Readiness and Motivation Questionnaire; SCARED, Screen for Child Anxiety Related Disorders; SRS(‐A), Social Responsiveness Scale (Adult).

*statistically significant association before application of False discovery rate correction (*p* < 0.05).

**statistically significant association after application of False discovery rate correction (q* < 0.0159); q* = corrected significance level by applying Benjamini‐Hochberg formula for False discovery rate.

Table 4A presents the results from the multiple regression analysis for RMQ precontemplation. To account for confounding effects due to multicollinearity, we only included the EDE total score (and not the separate EDE subscale scores) in the multiple regression analysis. In addition, the BDI and (C)Y‐BOCS total scores were included as other predictors. The overall regression was statistically significant (*R* ^2^ change = 0.54, *F*(4,59) = 16.69, *p* < 0.001) compared to a covariate‐only model. We found that the EDE total score significantly predicted the RMQ precontemplation score (beta = 0.61, *p* < 0.001). Age, (C)Y‐BOCS and BDI total score did not significantly contribute to the model. We repeated the multiple regression, excluding the EDE total score, and found that BDI made a significant contribution to the model ((*R* ^2^ change = 0.28, *F*(3,59) = 7.56, *p* < 0.001); beta = 0.48, *p* < 0.001) (Table 4B).

**TABLE 4A:** Predictors of RMQ precontemplation, EDE total score included.

Model	Predictor variables	B (95% CI)	Beta	*R* ^2^	*R* ^2^ change
1				0.01	0.01
	Age	0.69 (−1.91, 3.28)	0.07		
2				0.55	0.54***
	Age	−0.22 (−2.08, 1.65)	−0.02		
	BDI total score	0.27 (−0.13, 0.68)	0.15		
	EDE total score	11.18 (7.20, 15.16)	0.61***		
	(C)Y‐BOCS	0.22 (−0.18, 0.63)	0.11		

**TABLE 4B:** Predictors of RMQ precontemplation, EDE total score excluded.

Model	Predictor variables	B (95% CI)	Beta	*R* ^2^	*R* ^2^ change
1				0.01	0.01
	Age	0.69 (−1.91, 3.28)	0.07		
2				0.29	0.28***
	Age	−0.63 (−2.95, 1.68)	−0.06		
	BDI total score	0.84 (0.41, 1.28)	0.48***		
	(C)Y‐BOCS	0.32 (−0.19, 0.82)	0.15		

A multiple regression analysis for RMQ internality, where EDE total score and BDI were included as predictors, was also significant (*R* ^2^ change = 0.21, *F*(3,62) = 5.16, *p* < 0.01) compared to a covariate‐only model (Table 4C). EDE total score significantly predicted the RMQ internality score (beta = −0.32, *p* < 0.05). Age and BDI total scores did not significantly contribute to the prediction. For results excluding the EDE total score as predictor, we refer back to the linear regressions in Table 3B.

**TABLE 4C:** Predictors of RMQ internality.

Model	Predictor variables	B (95% CI)	Beta	*R* ^2^	*R* ^2^ change
1				0.00	0.00
	Age	−0.04 (−2.98, 2.90)	−0.00		
2				0.21	0.21**
	Age	0.68 (−2.06, 3.41)	0.06		
	BDI total score	−0.42 (−1.00, 0.16)	−0.20		
	EDE total score	−6.77 (−12.64, −0.89)	−0.32*		

*Note: R* ^2 change^ is based on original complete cases. All analyses were corrected for age at baseline.

Abbreviations: BDI, Beck Depression Inventory; (C)Y‐BOCS, (Children's) Yale Brown Obsessive‐Compulsive Scale; EDE, Eating Disorder Examination.

***p < 0.001, **p < 0.01, *p < 0.05.

No multiple regression analysis was conducted for RMQ confidence, as only one of the EDE subscale scores demonstrated an association after FDR correction.

### 3.4 The Association of Binge‐Eating, Self‐Induced Vomiting, and Laxative Misuse, With Motivation to Change

Participants with binge‐eating episodes had higher RMQ precontemplation scores, and participants with laxative misuse had higher RMQ action and RMQ confidence scores. These results are presented in Table 5.

**TABLE 5:** Comparison of RMQ scores between participants presenting with or without specific ED behaviours.

	Binge‐eating		Self‐induced vomiting		Laxative misuse	
	Present mean (SD)	Absent mean (SD)	Present mean (SD)	Absent mean (SD)	Present mean (SD)	Absent mean (SD)
RMQ precontemplation	72.98 (13.97)***	57.66 (23.61)***	72.53 (13.28)	61.10 (22.65)	67.47 (13.07)	62.03 (22.74)
RMQ action	30.93 (20.37)	32.07 (17.14)	39.55 (24.00)	30.83 (17.09)	44.58 (22.21)*	30.49 (17.26)*
RMQ internality	38.17 (23.45)	43.15 (25.89)	31.45 (29.31)	43.71 (24.67)	56.09 (26.54)	40.45 (25.01)
RMQ confidence	26.30 (17.19)	26.06 (15.85)	27.40 (20.14)	25.78 (15.62)	38.01 (20.30)*	24.58 (15.14)*

*Note:* We used the *p*‐value for ‘equal variances not assumed’ for the *t*‐test of the RMQ precontemplation score for participants with and without binge‐eating, because of a small violation of the Levene's test. For all other analyses, the assumption for equal variances was met.

Abbreviations: RMQ, Readiness and motivation questionnaire; SD, Standard deviation.

***p < 0.001, **p < 0.01, *p < 0.05 (2‐tailed).

After adjusting for EDE total score and age in an ANCOVA, the difference between the binge‐eating and the non‐binge‐eating group for RMQ precontemplation scores, became non‐significant (*F*(1,71) = 2.71, *p* = 0.104, partial *η* ^2^ = 0.04). Due to a small violation in the homogeneity of variances (*F*(1,73) = 4.155, *p* = 0.045), the results need to be interpreted with caution.

The difference between the group of participants that misused laxatives and the group that did not, remained significant for RMQ action (*F*(1,72) = 7.08, *p* = 0.01, partial *η* ^2^ = 0.09) and RMQ confidence (*F*(1,72) = 8.79, *p* = 0.004, partial *η* ^2^ = 0.11). According to Cohen's (1988) guidelines, these are medium effect sizes. Because of the exploratory design of this research aim, we chose not to apply FDR correction.

## 4 Discussion

The present study aimed to develop a more nuanced understanding of the patient‐related factors influencing motivation to change in a homogeneous adolescent first‐onset AN sample. Our findings indicated that adolescents with more severe ED symptoms, depressive symptoms, and obsessive‐compulsive symptoms, experienced lower motivation to change. The severity of ED symptoms emerged as the most important factor associated with motivation to change. Age, BMI‐SDS, anxiety symptoms, ASD‐symptoms, or ADHD‐symptoms were not associated with the degree of motivation to change. In addition, we investigated the association between specific ED behaviours (binge‐eating, self‐induced vomiting, and laxative misuse) and motivation to change; the findings indicated that engaging in laxative misuse was associated with more motivation to change. Binge‐eating was associated with lower motivation to change, however, this effect was attributed to the confounding effect of more severe ED symptoms.

Several previous studies in samples varying in age, illness duration and severity, and treatment setting (Hillen et al. 2015; McHugh 2007; Serrano et al. 2004; Rushford 2006; Ålgars et al. 2015; Zaitsoff and Taylor 2009), already found that individuals with AN who have more severe ED symptoms, showed less motivation to change. A similar association between ED symptoms and motivation to change emerges from our more homogeneous adolescent first‐onset AN sample. Moreover, when comparing our results to those from other studies using the RMQ we found that our sample obtained even lower motivational scores (Geller et al. 2013; Iyar et al. 2019). In our sample, adolescents with more severe ED symptoms were less ready to contemplate possible behaviour change, had lower internal motivation to make a behaviour change, and had less confidence in the ability to make a behaviour change. Specifically, we found that adolescents with more concerns about weight and body shape showed the least motivation to change. Similar results were found in another adolescent AN sample with a longer illness duration (Serrano et al. 2004). Together, these findings suggest that shape and weight concerns are the most significant ED symptom domains to be associated with motivation to change in adolescents with AN, both in female adolescents with a longer illness duration (Serrano et al. 2004) as well as those with a relatively short‐lived illness duration (this study).

Not only adolescents with more severe ED symptoms, but also adolescents with more severe depressive symptoms showed lower motivation to change in our sample, which replicates findings from previous research (McHugh 2007; Serrano et al. 2004). In addition, we found that adolescents with more severe obsessive‐compulsive symptoms showed lower motivation to change. This finding is novel to motivation research in AN as the association with obsessive‐compulsive symptoms has not previously been studied or reported. Part of the observed relationship of obsessive‐compulsive and depressive symptoms with motivation to change may be attributed to shared variance between the variables. However, when including all relevant patient‐related variables in one model, the severity of ED symptoms emerged as the dominant factor to be associated with motivation to change.

Contrary to our expectation, we did not find anxiety, ASD and ADHD symptoms to be associated with motivation to change in our sample of first‐onset adolescent AN. While the range of depressive, obsessive‐compulsive, and anxiety symptoms was quite broad in our sample and a substantial percentage of participants met the diagnostic classification for the relative disorders, this was less so for ASD and ADHD symptoms. This could explain, in part, why we did not find an association for ASD and ADHD symptoms with motivation to change. With regard to anxiety symptoms, a lack of association may be attributed to the fact that the anxiety symptoms—as measured by the SCARED—are not ED related and therefore do not influence ED related motivation to change, while the instruments measuring depressive and obsessive‐compulsive symptoms can have overlap with ED related symptomology.

We found no association between BMI‐SDS and motivation to change, in our predominantly outpatient sample. In contrast, previous clinical research in inpatient adolescents found an association between lower body weight and more motivation to change (Hillen et al. 2015; McHugh 2007). Inpatient treatment is generally reserved for AN patients with a severe impact of the ED on their body weight and physical health; BMI is therefore generally lower in an inpatient setting than in an outpatient setting. Furthermore, inpatient settings offer strict food rehabilitation schedules and focus on weekly weight gain, which puts more emphasis on body weight and weight gain during the course of treatment than in an outpatient setting, which in turn could influence the association between BMI and motivation. In addition, our sample consisted of adolescents with first‐onset AN with a short illness duration, it is also possible that in the initial stages of AN, this association is not yet present.

Adolescents who exhibited laxative misuse experienced more readiness to take action to change the ED. This was not the case for adolescents who experienced self‐induced vomiting or binge‐eating episodes. A previous study (Mond et al. 2006) found that laxative misuse was associated with both higher psychological distress and with lower physical wellbeing, but that self‐induced vomiting was associated with higher psychological distress only (not with lower physical wellbeing). These results, combined with results from our study, suggest that experiencing lower physical wellbeing might be associated with more readiness to actively change their ED. However, results should be interpreted with caution, as only few participants reported exhibiting these behaviours.

### 4.1 Strengths and Limitations

To the best of our knowledge, this is the first study that investigated motivation to change in a large homogeneous adolescent first‐onset AN sample. Additionally, it included many predictor variables in the same study, which allowed for direct comparison of a wide variety of symptoms. To our knowledge, no previous study reported on the association of obsessive‐compulsive, ASD, and ADHD symptoms, with motivation to change. This is also the first study to report on the internal consistency of the Dutch translation of the RMQ.

There are several limitations to the findings reported in this study as well. First, the cross‐sectional design of our study does not allow any conclusions regarding causal relationships between the investigated variables. Second, several comorbidity measures had missing data, and since these missing values were associated with more severe ED symptoms, this may result in an underrepresentation of cases with more severe AN. Third, this study focused on patient variables related to type and severity of AN symptoms and comorbidity, which left other patient‐related factors such as personality traits, illness coherence, emotional and physical well‐being, and context‐related factors such as life events and relationships with important others, out of scope. We can also not exclude that shared rater bias variance may increase the relationship in some variables, however, since several of the measures that were collected were interviews or parent rating scales, our key findings are not affected by this type of bias.

### 4.2 Implications for Clinical Practice

Our findings and those of previous research provide support for the hypothesis that motivational dynamics are influenced by ED type, age, weight, illness severity and duration, treatment setting, degree of concern about body weight and body shape, and obsessive‐compulsive and depressive symptoms. As motivation to change is an important component in understanding and improving therapeutic outcomes (Vall and Wade 2015), being mindful of these characteristics and symptoms can prove to be an important tool in personalising AN treatment.

Previous research already demonstrated that early treatment is associated with improved ED outcomes (Mills, Hyam, and Schmidt 2024). These improved outcomes might not only be mediated by early ED symptom reduction, but also by the positive effects that early symptom reduction has on improving adolescents' capacity to consider potential behaviour changes and to take action toward modifying their ED.

As the presence of depressive symptoms appears to have an additional negative effect on motivation to change in adolescents with first‐onset AN—regardless of the effect that is exerted by the severity of ED symptoms—being attentive to the presence of depressive symptoms during treatment may enhance motivation to change, and through such potentially improve the course and outcome of AN.

The use of the Dutch translation of the RMQ in our study contributes to the possible application of this instrument in the Dutch clinical care and therefore offers an alternative instrument to measure motivation to change that is suitable for younger populations, and that is aligned with the EDE, the golden standard for measuring ED symptoms.

### 4.3 Implications for Future Research

This study contributes to the existing body of literature by investigating the combined effect of a multitude of potential predictors of motivation to change within the same sample. The severity and type of ED symptoms were significantly associated with motivation to change, suggesting that future research focusing on a comprehensive examination of the relationship between motivation to change and ED symptoms may yield valuable insights. The RMQ, with different motivational scales available for different ED symptom domains, is well suited as an instrument to explore this relationship.

Future research examining the relationship between ED symptoms and broader psychological, relational and contextual factors, would add valuable information to the field of motivational research. Lastly, replication of our results would provide further evidence to support our findings.

## Conflicts of Interest

The authors declare no conflicts of interest.

## Permission to Reproduce Material From Other Sources

The developer of the Readiness and Motivation Questionnaire (RMQ; Geller et al. 2013) granted us permission to use the instrument and to share it in the appendix. Researchers interested in using the instrument can contact the developer Dr. Josie Geller: jgeller@providencehealth.bc.ca. Researchers interested in using the Dutch version of the RMQ can contact Dr. Gwendolyn C. Dieleman: g.dieleman@erasmusmc.nl.

## Supporting information

Supporting Information S1

## Data Availability

Data access and requests for collaboration are welcome, and will be conducted in accordance with the European Union's General Data Protection Regulation (GDPR).
