# Supplementary material for: Patient‐Related Factors Influencing Motivation to Change in Adolescents With First‐Onset Anorexia Nervosa: A Cross‐Sectional Study
Source: Eur Eat Disord Rev. 2025 Feb 15;33(4):763–75. doi: 10.1002/erv.3182 (PMC12171662; doi:10.1002/erv.3182)
Supplement: Supplementary file 1 — Supporting Information S1 [file ERV-33-763-s001.docx]

**APPENDIX 1**

Readiness and Motivation Questionnaire (RMQ)

**1. DIETARY RESTRICTION**

**ON HOW MANY DAYS OUT OF THE PAST 28 DAYS have you consciously tried to restrict the amount of food you eat to influence shape or weight?** (please circle one of the following)

None 1-5 Days 6-12 Days 13-15 Days 16-22 Days 23-27 Days Daily

*If you have* ***never tried to restrict****, please go to the next section.*

*If you have* ***ever restricted****, please answer the following questions:*

**IN THE PAST TWO WEEKS, how much of you has wanted to restrict your eating?** (please circle one of the following percentages)

A small part of me About half of me Most of me

0% 10 20 30 40 50 60 70 80 90 100%

**IN THE PAST TWO WEEKS, how much of you has been actively working to eat more?** (please circle one of the following percentages)

A small part of me About half of me Most of me

0% 10 20 30 40 50 60 70 80 90 100%

**If you were to reduce your restriction (i.e. eat more), how much of this would be for you (versus for others)?** (please circle one of the following percentages)

Not very much for me About half for me Mostly for me

0% 10 20 30 40 50 60 70 80 90 100%

**If you decided to reduce your restriction (i.e., eat more), how confident are you in your ability to do so?** (please circle one of the following percentages)

Not at all confident Extremely confident

0% 10 20 30 40 50 60 70 80 90 100%

**2.1 MAINTAINING LOW WEIGHT (only answer if you are currently underweight)**

**ON HOW MANY DAYS OUT OF THE PAST 28 DAYS have you consciously tried to maintain a low weight?** (please circle one of the following)

None 1-5 Days 6-12 Days 13-15 Days 16-22 Days 23-27 Days Daily

*If you have* ***never tried to maintain a low weight****, please go to the next section.*

*If you have* ***ever tried to maintain a low weight****, please answer the following questions:*

**IN THE PAST TWO WEEKS, how much of you has wanted to maintain a low weight?** (please circle one of the following percentages)

A small part of me About half of me Most of me

0% 10 20 30 40 50 60 70 80 90 100%

**IN THE PAST TWO WEEKS, how much of you has been actively working to gain weight?** (please circle one of the following percentages)

A small part of me About half of me Most of me

0% 10 20 30 40 50 60 70 80 90 100%

**If you were to gain weight, how much of this would be for you (versus for others)?** (please circle one of the following percentages)

Not very much for me About half for me Mostly for me

0% 10 20 30 40 50 60 70 80 90 100%

**If you decided to gain weight, how confident are you in your ability to do so?** (please circle one of the following percentages)

Not at all confident Extremely confident

0% 10 20 30 40 50 60 70 80 90 100%

**2.2 WEIGHT LOSS (only answer if you responded “none” to the previous question)**

**ON HOW MANY DAYS OUT OF THE PAST 28 DAYS have you tried to lose weight?** (please circle one of the following)

None 1-5 Days 6-12 Days 13-15 Days 16-22 Days 23-27 Days Daily

*If you have* ***never tried to lose weight****, please go to the next section.*

*If you have* ***ever tried to lose weight****, please answer the following questions:*

**IN THE PAST TWO WEEKS, how much of you has wanted to lose weight?** (please circle one of the following percentages)

A small part of me About half of me Most of me

0% 10 20 30 40 50 60 70 80 90 100%

**IN THE PAST TWO WEEKS, how much of you has been actively working to stop your efforts to lose weight?** (please circle one of the following percentages)

A small part of me About half of me Most of me

0% 10 20 30 40 50 60 70 80 90 100%

**If you were to stop your efforts to lose weight how much of this would be for you (versus for others)?** (please circle one of the following percentages)

Not very much for me About half for me Mostly for me

0% 10 20 30 40 50 60 70 80 90 100%

**If you decided to stop your efforts to lose weight, how confident are you in your ability to do so?** (please circle one of the following percentages)

Not at all confident Extremely confident

0% 10 20 30 40 50 60 70 80 90 100%

**3. MENSTRUATION**

**Are you on the birth control pill?** YES NO

**Have you missed any periods over the past three months?** YES NO

**If yes, how many periods have you missed?____________**

*If you answered no:* **Have you ever missed any periods due to weight loss?** YES NO

*If you have* ***never missed your period****, please go to the next section.*

*If you have* ***ever missed your period due to weight loss****, please answer the following questions:*

**IN THE PAST TWO WEEKS, how much of you has not wanted to have periods?** (please circle one of the following percentages)

A small part of me About half of me Most of me

0% 10 20 30 40 50 60 70 80 90 100%

**IN THE PAST TWO WEEKS, how much of you has been actively working to get your periods back?** (please circle one of the following percentages)

A small part of me About half of me Most of me

0% 10 20 30 40 50 60 70 80 90 100%

**If you were to try to get your periods back, how much of this would be for you (versus for others)?** (please circle one of the following percentages)

Not very much for me About half for me Mostly for me

0% 10 20 30 40 50 60 70 80 90 100%

**If you decided to try to get your periods back, how confident are you in your ability to do so?** (please circle one of the following percentages)

Not at all confident Extremely confident

0% 10 20 30 40 50 60 70 80 90 100%

**4. EPISODES OF OVEREATING (i.e. Bingeing)**

**ON HOW MANY DAYS OUT OF THE PAST 28 DAYS have you eaten an objectively large amount of food and most people would agree it was too much to have at one time, (i.e. bingeing) accompanied by a loss of control (i.e. you couldn't resist eating, or once you started you felt that you couldn't stop)?** (please circle one of the following)

None 1-5 Days 6-12 Days 13-15 Days 16-22 Days 23-27 Days Daily

*If you have* ***never binged****, please go to the next section.*

*If you have* ***ever binged,*** *please answer the following questions:*

**IN THE PAST TWO WEEKS, how much of you has wanted to binge?** (please circle one of the following percentages)

A small part of me About half of me Most of me

0% 10 20 30 40 50 60 70 80 90 100%

**IN THE PAST TWO WEEKS, how much of you has been actively working to reduce your bingeing?** (please circle one of the following percentages)

A small part of me About half of me Most of me

0% 10 20 30 40 50 60 70 80 90 100%

**If you were to reduce your bingeing, how much of this would be for you (versus for others)?** (please circle one of the following percentages)

Not very much for me About half for me Mostly for me

0% 10 20 30 40 50 60 70 80 90 100%

**If you decided to reduce your bingeing, how confident are you in your ability to do so?** (please circle one of the following percentages)

Not at all confident Extremely confident

0% 10 20 30 40 50 60 70 80 90 100%

**5. DIETARY RESTRICTION OUTSIDE EPISODES OF OVEREATING** *Only answer this question if you answered yes to question 4***

**ON HOW MANY DAYS OUT OF THE PAST 28 DAYS have you restricted your eating (i.e. < 1200 calories/day) before or after binges in order to compensate for the binge?** (please circle one of the following)

None 1-5 Days 6-12 Days 13-15 Days 16-22 Days 23-27 Days Daily

*If you have* ***never tried to restrict to compensate for bingeing****, please go to the next section.*

*If you have* ***ever restricted to compensate for bingeing,*** *please answer the following questions:*

**IN THE PAST TWO WEEKS, how much of you has wanted to restrict to compensate for binges?** (please circle one of the following percentages)

A small part of me About half of me Most of me

0% 10 20 30 40 50 60 70 80 90 100%

**IN THE PAST TWO WEEKS, how much of you has been actively working to reduce your restriction before or after a binge?** (please circle one of the following percentages)

A small part of me About half of me Most of me

0% 10 20 30 40 50 60 70 80 90 100%

**If you were to reduce your restriction before or after a binge, how much of this would be for you (versus for others?)** (please circle one of the following percentages)

Not very much for me About half for me Mostly for me

0% 10 20 30 40 50 60 70 80 90 100%

**If you decided to reduce your restriction before or after a binge, how confident are you in your ability to do so?** (please circle one of the following percentages)

Not at all confident Extremely confident

0% 10 20 30 40 50 60 70 80 90 100%

**6. SELF INDUCED VOMITING**

**ON HOW MANY DAYS OUT OF THE PAST 28 DAYS have you made yourself sick (vomit) as a means of controlling your shape or weight or to counteract the effects of eating?** (please circle one of the following)

None 1-5 Days 6-12 Days 13-15 Days 16-22 Days 23-27 Days Daily

*If you have* ***never made yourself sick,*** *please go to the next section.*

*If you have* ***ever made yourself sick,*** *please answer the following questions:*

**IN THE PAST TWO WEEKS, how much of you has wanted to make yourself sick?** (please circle one of the following percentages)

A small part of me About half of me Most of me

0% 10 20 30 40 50 60 70 80 90 100%

**IN THE PAST TWO WEEKS, how much of you has been actively working to reduce the amount you make yourself sick?** (please circle one of the following percentages)

A small part of me About half of me Most of me

0% 10 20 30 40 50 60 70 80 90 100%

**If you were to reduce the amount you make yourself sick, how much of this would be for you (versus for others)?** (please circle one of the following percentages)

Not very much for me About half for me Mostly for me

0% 10 20 30 40 50 60 70 80 90 100%

**If you decided to reduce the amount you make yourself sick, how confident are you in your ability to do so?** (please circle one of the following percentages)

Not at all confident Extremely confident

0% 10 20 30 40 50 60 70 80 90 100%

**7. LAXATIVE MISUSE**

**ON HOW MANY DAYS OUT OF THE PAST 28 DAYS have you taken laxatives as a means of controlling your shape or weight or to counteract the effects of eating?** (please circle one of the following)

None 1-5 Days 6-12 Days 13-15 Days 16-22 Days 23-27 Days Daily

*If you have* ***never misused laxatives,*** *please go to the next section.*

*If you have* ***ever misused laxatives,*** *please answer the following questions:*

**IN THE PAST TWO WEEKS, how much of you has wanted to use laxatives to control shape/weight?** (please circle one of the following percentages)

A small part of me About half of me Most of me

0% 10 20 30 40 50 60 70 80 90 100%

**IN THE PAST TWO WEEKS, how much of you has been actively working to reduce your laxative use?** (please circle one of the following percentages)

A small part of me About half of me Most of me

0% 10 20 30 40 50 60 70 80 90 100%

**If you were to reduce your laxative use, how much of this would be for you (versus for others)?** (please circle one of the following percentages)

Not very much for me About half for me Mostly for me

0% 10 20 30 40 50 60 70 80 90 100%

**If you decided to reduce your laxative use, how confident are you in your ability to do so?** (please circle one of the following percentages)

Not at all confident Extremely confident

0% 10 20 30 40 50 60 70 80 90 100%

**8. DIURETIC MISUSE**

**ON HOW MANY DAYS OUT OF THE PAST 28 DAYS have you taken diuretics (water tablets) as a means of controlling your shape or weight or to counteract the effects of eating?** (please circle one of the following)

None 1-5 Days 6-12 Days 13-15 Days 16-22 Days 23-27 Days Daily

*If you have* ***never misused diuretics,*** *please go to the next section.*

*If you have* ***ever misused diuretics,*** *please answer the following questions:*

**IN THE PAST TWO WEEKS, how much of you has wanted to use diuretics to control shape/weight?** (please circle one of the following percentages)

A small part of me About half of me Most of me

0% 10 20 30 40 50 60 70 80 90 100%

**IN THE PAST TWO WEEKS, how much of you has been actively working to reduce your diuretics use?** (please circle one of the following percentages)

A small part of me About half of me Most of me

0% 10 20 30 40 50 60 70 80 90 100%

**If you were to reduce your diuretics use, how much of this would be for you (versus for others)?** (please circle one of the following percentages)

Not very much for me About half for me Mostly for me

0% 10 20 30 40 50 60 70 80 90 100%

**If you decided to reduce your diuretics use, how confident are you in your ability to do so?** (please circle one of the following percentages)

Not at all confident Extremely confident

0% 10 20 30 40 50 60 70 80 90 100%

**9. EXERCISE TO CONTROL SHAPE OR WEIGHT**

**ON HOW MANY DAYS OUT OF THE PAST 28 DAYS have you vigorously exercised as a means of controlling your weight, altering your shape or amount of fat, or burning off calories?** (please circle one of the following)

None 1-5 Days 6-12 Days 13-15 Days 16-22 Days 23-27 Days Daily

*If you have* ***never exercised to control shape or weight,*** *please go to the next section.*

*If you have* ***ever exercised to control shape or weight,*** *please answer the following questions:*

**IN THE PAST TWO WEEKS, how much of you has wanted to exercise?** (please circle one of the following percentages)

A small part of me About half of me Most of me

0% 10 20 30 40 50 60 70 80 90 100%

**IN THE PAST TWO WEEKS, how much of you has been actively working to reduce your exercise?** (please circle one of the following percentages)

A small part of me About half of me Most of me

0% 10 20 30 40 50 60 70 80 90 100%

**If you were to reduce your exercise, how much of this would be for you (versus for others)?** (please circle one of the following percentages)

Not very much for me About half for me Mostly for me

0% 10 20 30 40 50 60 70 80 90 100%

**If you decided to reduce your exercise, how confident are you in your ability to do so?** (please circle one of the following percentages)

Not at all confident Extremely confident

0% 10 20 30 40 50 60 70 80 90 100%

**10. FEAR OF WEIGHT GAIN**

**ON HOW MANY DAYS OUT OF THE PAST 28 DAYS have you had a definite fear that you might gain weight or become fat?** (please circle one of the following)

None 1-5 Days 6-12 Days 13-15 Days 16-22 Days 23-27 Days Daily

*If you have* ***never had a fear of weight gain,*** *please go to the next section.*

*If you have* ***ever had a fear of weight gain,*** *please answer the following questions:*

**IN THE PAST TWO WEEKS, how much of you has wanted to hold on to your fear of weight gain?** (please circle one of the following percentages)

A small part of me About half of me Most of me

0% 10 20 30 40 50 60 70 80 90 100%

**IN THE PAST TWO WEEKS, how much of you has been actively working to reduce your fear of weight gain?** (please circle one of the following percentages)

A small part of me About half of me Most of me

0% 10 20 30 40 50 60 70 80 90 100%

**If you were to reduce your fear of weight gain, how much of this would be for you (versus for others)?** (please circle one of the following percentages)

Not very much for me About half for me Mostly for me

0% 10 20 30 40 50 60 70 80 90 100%

**If you decided to reduce your fear of weight gain, how confident are you in your ability to do so?** (please circle one of the following percentages)

Not at all confident Extremely confident

0% 10 20 30 40 50 60 70 80 90 100%

**11. FEELINGS OF FATNESS**

**ON HOW MANY DAYS OUT OF THE PAST 28 DAYS have you felt fat?** (please circle one of the following)

None 1-5 Days 6-12 Days 13-15 Days 16-22 Days 23-27 Days Daily

*If you have* ***never felt fat,*** *please go to the next section.*

*If you have* ***ever felt fat,*** *please answer the following questions:*

**IN THE PAST TWO WEEKS, how much of you has wanted to hold on to feelings of fatness?** (please circle one of the following percentages)

A small part of me About half of me Most of me

0% 10 20 30 40 50 60 70 80 90 100%

**IN THE PAST TWO WEEKS, how much of you has been actively working to reduce your feelings of fatness?** (please circle one of the following percentages)

A small part of me About half of me Most of me

0% 10 20 30 40 50 60 70 80 90 100%

**If you were to reduce your feelings of fatness?, how much of this would be for you (versus for others)?** (please circle one of the following percentages)

Not very much for me About half for me Mostly for me

0% 10 20 30 40 50 60 70 80 90 100%

**If you decided to reduce your feelings of fatness?, how confident are you in your ability to do so?** (please circle one of the following percentages)

Not at all confident Extremely confident

0% 10 20 30 40 50 60 70 80 90 100%

**12. IMPORTANCE OF SHAPE AND WEIGHT**

**ON HOW MANY DAYS OUT OF THE PAST 28 DAYS has your weight and/or shape influenced how you think about (judge) yourself as a person?** (please circle one of the following)

None 1-5 Days 6-12 Days 13-15 Days 16-22 Days 23-27 Days Daily

*If shape or weight have* ***never influenced how you judge yourself,*** *please go to the next section.*

*If shape or weight have* ***ever influenced how you judge yourself,*** *please answer the following questions:*

**IN THE PAST TWO WEEKS, how much of you has wanted your shape or weight to matter?** (please circle one of the following percentages)

A small part of me About half of me Most of me

0% 10 20 30 40 50 60 70 80 90 100%

**IN THE PAST TWO WEEKS, how much of you has been actively working to make shape or weight matter less?** (please circle one of the following percentages)

A small part of me About half of me Most of me

0% 10 20 30 40 50 60 70 80 90 100%

**If you were to try to make shape or weight matter less, how much of this would be for you (versus for others)?** (please circle one of the following percentages)

Not very much for me About half for me Mostly for me

0% 10 20 30 40 50 60 70 80 90 100%

**If you decided to make shape and weight matter less, how confident are you in your ability to do so?** (please circle one of the following percentages)

Not at all confident Extremely confident

0% 10 20 30 40 50 60 70 80 90 100%

**APPENDIX 2**

Readiness and Motivation Questionnaire - Dutch (RMQ-Dutch)

**1. BEPERKING VAN DE VOEDSELINNAME**

**Op hoeveel van de afgelopen 28 dagen heb jij bewust geprobeerd om je voedselinname te beperken om je lichaamsvorm of gewicht te beïnvloeden?** (Omcirkel een van de antwoordmogelijkheden)

Geen 1-5 Dagen 6-12 Dagen 13-15 Dagen 16-22 Dagen 23-27 Dagen Dagelijks

*Als je* ***nooit jezelf hebt opgelegd om minder te eten****, ga dan verder met de volgende sectie.*

*Als je jezelf* ***hebt opgelegd om minder te eten****, geef dan antwoord op de volgende vragen:*

**In de afgelopen TWEE weken, hoe groot was het deel van jou dat minder wilde eten?** (omcirkel een van de volgende percentages)

Een klein deel van mij Ongeveer de helft van mij Het grootste deel van mij

0% 10 20 30 40 50 60 70 80 90 100%

**In de afgelopen TWEE weken, hoe groot was het deel van jou dat bewust probeerde om je voedselinname te vergroten?** (omcirkel een van de volgende percentages)

Een klein deel van mij Ongeveer de helft van mij Het grootste deel van mij

0% 10 20 30 40 50 60 70 80 90 100%

**Stel dat je probeert om je voedselinname minder te beperken (dat wil zeggen meer te eten), in hoeverre doe je dit dan voor jezelf (in tegenstelling tot voor anderen)?** (omcirkel een van de volgende percentages)

Nauwelijks voor mij Ongeveer half voor mij Vooral voor mij

0% 10 20 30 40 50 60 70 80 90 100%

**Stel dat je besluit om je voedselinname minder te beperken (dat wil zeggen meer te eten), hoe zeker ben je dan dat je dat lukt?** (omcirkel een van de volgende percentages)

Helemaal niet zeker Heel zeker

0% 10 20 30 40 50 60 70 80 90 100%

**2.1 HET BEHOUDEN VAN EEN LAAG GEWICHT (geef alleen antwoord op deze vragen wanneer jij op dit moment een ondergewicht hebt)**

**Op hoeveel van de afgelopen 28 dagen heb jij geprobeerd een laag gewicht te behouden?** (Omcirkel een van de antwoordmogelijkheden)

Geen 1-5 Dagen 6-12 Dagen 13-15 Dagen 16-22 Dagen 23-27 Dagen Dagelijks

*Als je* ***nooit hebt geprobeerd om een laag gewicht te behouden****, ga dan verder met de volgende sectie.*

*Als je* ***ooit hebt geprobeerd om een laag gewicht te behouden****, geef dan antwoord op de volgende vragen:*

**In de afgelopen TWEE weken, hoe groot was het deel van jou dat een laag gewicht wilde behouden?** (omcirkel een van de volgende percentages)

Een klein deel van mij Ongeveer de helft van mij Het grootste deel van mij

0% 10 20 30 40 50 60 70 80 90 100%

**In de afgelopen TWEE weken, hoe groot was het deel van jou dat bewust probeerde om aan te komen in gewicht?** (omcirkel een van de volgende percentages)

Een klein deel van mij Ongeveer de helft van mij Het grootste deel van mij

0% 10 20 30 40 50 60 70 80 90 100%

**Stel dat je probeert om aan te komen, in hoeverre doe je dit dan voor jezelf (in tegenstelling tot voor anderen)?** (omcirkel een van de volgende percentages)

Nauwelijks voor mij Ongeveer half voor mij Vooral voor mij

0% 10 20 30 40 50 60 70 80 90 100%

**Stel dat je besluit om aan te komen, hoe zeker ben je dan dat je dat lukt?** (omcirkel een van de volgende percentages)

Helemaal niet zeker Heel zeker

0% 10 20 30 40 50 60 70 80 90 100%

**2.2 HET VERLIEZEN VAN GEWICHT (geef alleen antwoord op deze vragen wanneer jij de vorige vraag “geen” hebt geantwoord)**

**Op hoeveel van de afgelopen 28 dagen heb jij geprobeerd om gewicht te verliezen?** (Omcirkel een van de antwoordmogelijkheden)

Geen 1-5 Dagen 6-12 Dagen 13-15 Dagen 16-22 Dagen 23-27 Dagen Dagelijks

*Als je* ***nooit hebt geprobeerd om gewicht te verliezen****, ga dan verder met de volgende sectie.*

*Als je* ***ooit hebt geprobeerd om gewicht te verliezen****, geef dan antwoord op de volgende vragen:*

**In de afgelopen TWEE weken, hoe groot was het deel van jou dat gewicht wilde verliezen?** (omcirkel een van de volgende percentages)

Een klein deel van mij Ongeveer de helft van mij Het grootste deel van mij

0% 10 20 30 40 50 60 70 80 90 100%

**In de afgelopen TWEE weken, hoe groot was het deel van jou dat bewust probeerde om het verliezen van gewicht te stoppen?** (omcirkel een van de volgende percentages)

Een klein deel van mij Ongeveer de helft van mij Het grootste deel van mij

0% 10 20 30 40 50 60 70 80 90 100%

**Stel dat je probeert om het verliezen van gewicht te stoppen, in hoeverre doe je dit dan voor jezelf (in tegenstelling tot voor anderen)?** (omcirkel een van de volgende percentages)

Nauwelijks voor mij Ongeveer half voor mij Vooral voor mij

0% 10 20 30 40 50 60 70 80 90 100%

**Stel dat je besluit om het verliezen van gewicht te stoppen, hoe zeker ben je dan dat je dat lukt?** (omcirkel een van de volgende percentages)

Helemaal niet zeker Heel zeker

0% 10 20 30 40 50 60 70 80 90 100%

**3. MENSTRUATIE**

**Slik je de anticonceptiepil?** JA NEE

**Is jouw menstruatie in de afgelopen 3 maanden één of meerdere keren uitgebleven?** JA NEE

**Zo ja, hoe vaak is je menstruatie uitgebleven? ____________**keren

*Als je nee hebt geantwoord:* **Is je menstruatie ooit uitgebleven als gevolg van gewichtverlies?** JA NEE

*Als je* ***nooit hebt meegemaakt dat je menstruatie is uitgebleven als gevolg van gewichtverlies****, ga dan verder met de volgende sectie.*

*Als je* ***ooit hebt meegemaakt dat je menstruatie is uitgebleven als gevolg van gewichtverlies****, geef dan antwoord op de volgende vragen:*

**In de afgelopen TWEE weken, hoe groot was het deel van jou dat niet wilde menstrueren?** (omcirkel een van de volgende percentages)

Een klein deel van mij Ongeveer de helft van mij Het grootste deel van mij

0% 10 20 30 40 50 60 70 80 90 100%

**In de afgelopen TWEE weken, hoe groot was het deel van jou dat bewust probeerde om jouw menstruatiecyclus weer op gang te brengen** (omcirkel een van de volgende percentages)

Een klein deel van mij Ongeveer de helft van mij Het grootste deel van mij

0% 10 20 30 40 50 60 70 80 90 100%

**Stel dat je probeert om jouw menstruatiecyclus weer op gang te brengen, in hoeverre doe je dit dan voor jezelf (in tegenstelling tot voor anderen)?** (omcirkel een van de volgende percentages)

Nauwelijks voor mij Ongeveer half voor mij Vooral voor mij

0% 10 20 30 40 50 60 70 80 90 100%

**Stel dat je besluit om je menstruatiecyclus weer op gang te brengen, hoe zeker ben je dan dat je dat lukt?** (omcirkel een van de volgende percentages)

Helemaal niet zeker Heel zeker

0% 10 20 30 40 50 60 70 80 90 100%

**4. PERIODES VAN EETBUIEN**

**Op hoeveel van de afgelopen 28 dagen heb je zoveel gegeten dat de meeste mensen het erover eens zouden zijn dat het te veel was om in een keer te eten (eetbui), zonder dat je er controle over had? (dat wil zeggen je kon geen weestand bieden aan het eten of je had het gevoel dat je niet kon stoppen toen je eenmaal begonnen was)** (Omcirkel een van de antwoordmogelijkheden)

Geen 1-5 Dagen 6-12 Dagen 13-15 Dagen 16-22 Dagen 23-27 Dagen Dagelijks

*Als je* ***nooit een eetbui hebt gehad****, ga dan verder met de volgende sectie.*

*Als je* ***ooit een eetbui hebt gehad****, geef dan antwoord op de volgende vragen:*

**In de afgelopen TWEE weken, hoe groot was het deel van jou dat grote hoeveelheden wilde eten?** (omcirkel een van de volgende percentages)

Een klein deel van mij Ongeveer de helft van mij Het grootste deel van mij

0% 10 20 30 40 50 60 70 80 90 100%

**In de afgelopen TWEE weken, hoe groot was het deel van jou dat bewust probeerde om het aantal eetbuien te verminderen?** (omcirkel een van de volgende percentages)

Een klein deel van mij Ongeveer de helft van mij Het grootste deel van mij

0% 10 20 30 40 50 60 70 80 90 100%

**Stel dat je probeert om het aantal eetbuien te verminderen, in hoeverre doe je dit dan voor jezelf (in tegenstelling tot voor anderen)?** (omcirkel een van de volgende percentages)

Nauwelijks voor mij Ongeveer half voor mij Vooral voor mij

0% 10 20 30 40 50 60 70 80 90 100%

**Stel dat je besluit om het aantal eetbuien te verminderen, hoe zeker ben je dan dat je dat lukt?** (omcirkel een van de volgende percentages)

Helemaal niet zeker Heel zeker

0% 10 20 30 40 50 60 70 80 90 100%

**5. BEPERKEN VAN DE HOEVEELHEID ETEN BUITEN PERIODES VAN EETBUIEN *(geef alleen antwoord op deze vragen wanneer je vraag 4 positief hebt beantwoord)***

**Op hoeveel van de afgelopen 28 dagen heb jij minder gegeten (dat wil zeggen minder dan 1200 calorieën/dag) voor of na een eetbui om te compenseren voor de eetbui?** (Omcirkel een van de antwoordmogelijkheden)

Geen 1-5 Dagen 6-12 Dagen 13-15 Dagen 16-22 Dagen 23-27 Dagen Dagelijks

*Als je* ***nooit hebt geprobeerd om te compenseren voor een eetbui,*** *ga dan verder met de volgende sectie.*

*Als je* ***ooit een eetbui hebt gehad****, geef dan antwoord op de volgende vragen:*

**In de afgelopen TWEE weken, hoe groot was het deel van jou dat *minder of niet wilde eten* om te compenseren voor eetbuien?** (omcirkel een van de volgende percentages)

Een klein deel van mij Ongeveer de helft van mij Het grootste deel van mij

0% 10 20 30 40 50 60 70 80 90 100%

**In de afgelopen TWEE weken, hoe groot was het deel van jou dat bewust probeerde om het *minder of niet eten* voor of na een eetbui te verminderen?** (omcirkel een van de volgende percentages)

Een klein deel van mij Ongeveer de helft van mij Het grootste deel van mij

0% 10 20 30 40 50 60 70 80 90 100%

**Stel dat je probeert om het *minder of niet eten* voor of na een eetbui te verminderen, in hoeverre doe je dit dan voor jezelf (in tegenstelling tot voor anderen)?** (omcirkel een van de volgende percentages)

Nauwelijks voor mij Ongeveer half voor mij Vooral voor mij

0% 10 20 30 40 50 60 70 80 90 100%

**Stel dat je besluit om *niet minder te eten* voor of na een eetbui, hoe zeker ben je dan dat je dat lukt?** (omcirkel een van de volgende percentages)

Helemaal niet zeker Heel zeker

0% 10 20 30 40 50 60 70 80 90 100%

**6. ZELFOPGEWEKT BRAKEN**

**Op hoeveel van de afgelopen 28 dagen heb jij met opzet gebraakt als middel om je lichaamsvorm of gewicht onder controle te houden of om tegen de effecten van eten in te gaan?** (Omcirkel een van de antwoordmogelijkheden)

Geen 1-5 Dagen 6-12 Dagen 13-15 Dagen 16-22 Dagen 23-27 Dagen Dagelijks

*Als je* ***nooit hebt geprobeerd met opzet te braken,*** *ga dan verder met de volgende sectie.*

*Als je* ***ooit hebt geprobeerd met opzet te braken****, geef dan antwoord op de volgende vragen:*

**In de afgelopen TWEE weken, hoe groot was het deel van jou dat met opzet wilde braken?** (omcirkel een van de volgende percentages)

Een klein deel van mij Ongeveer de helft van mij Het grootste deel van mij

0% 10 20 30 40 50 60 70 80 90 100%

**In de afgelopen TWEE weken, hoe groot was het deel van jou dat bewust probeerde om minder vaak met opzet te braken?** (omcirkel een van de volgende percentages)

Een klein deel van mij Ongeveer de helft van mij Het grootste deel van mij

0% 10 20 30 40 50 60 70 80 90 100%

**Stel dat je probeert om minder vaak met opzet te braken, in hoeverre doe je dit dan voor jezelf (in tegenstelling tot voor anderen)?** (omcirkel een van de volgende percentages)

Nauwelijks voor mij Ongeveer half voor mij Vooral voor mij

0% 10 20 30 40 50 60 70 80 90 100%

**Stel dat je besluit om minder vaak met opzet te braken, hoe zeker ben je dan dat je dat lukt?** (omcirkel een van de volgende percentages)

Helemaal niet zeker Heel zeker

0% 10 20 30 40 50 60 70 80 90 100%

**7. LAXEERMIDDELEN**

**Op hoeveel van de afgelopen 28 dagen heb jij laxeermiddelen gebruikt als middel om je lichaamsvorm of gewicht onder controle te houden of om tegen de effecten van eten in te gaan?** (Omcirkel een van de antwoordmogelijkheden)

Geen 1-5 Dagen 6-12 Dagen 13-15 Dagen 16-22 Dagen 23-27 Dagen Dagelijks

*Als je* ***nooit laxeermiddelen hebt gebruik als middel om je lichaamsvorm/gewicht onder controle te houden,*** *ga dan verder met de volgende sectie.*

*Als je* ***ooit laxeermiddelen hebt gebruik als middel om je lichaamsvorm/gewicht onder controle te houden****, geef dan antwoord op de volgende vragen:*

**In de afgelopen TWEE weken, hoe groot was het deel van jou dat laxeermiddelen wilde gebruiken als middel om jouw lichaamsvorm/gewicht te beheersen?** (omcirkel een van de volgende percentages)

Een klein deel van mij Ongeveer de helft van mij Het grootste deel van mij

0% 10 20 30 40 50 60 70 80 90 100%

**In de afgelopen TWEE weken, hoe groot was het deel van jou dat bewust probeerde om het gebruik van laxeermiddelen te verminderen?** (omcirkel een van de volgende percentages)

Een klein deel van mij Ongeveer de helft van mij Het grootste deel van mij

0% 10 20 30 40 50 60 70 80 90 100%

**Stel dat je probeert om het gebruik van laxeermiddelen te verminderen, in hoeverre doe je dit dan voor jezelf (in tegenstelling tot voor anderen)?** (omcirkel een van de volgende percentages)

Nauwelijks voor mij Ongeveer half voor mij Vooral voor mij

0% 10 20 30 40 50 60 70 80 90 100%

**Stel dat je besluit om het gebruik van laxeermiddelen te verminderen, hoe zeker ben je dan dat je dat lukt?** (omcirkel een van de volgende percentages)

Helemaal niet zeker Heel zeker

0% 10 20 30 40 50 60 70 80 90 100%

**8. DIURETICA (PLASTABLETTEN)**

**Op hoeveel van de afgelopen 28 dagen heb jij diuretica (plastabletten) gebruikt als middel om je lichaamsvorm of gewicht onder controle te houden of om tegen de effecten van eten in te gaan?** (Omcirkel een van de antwoordmogelijkheden)

Geen 1-5 Dagen 6-12 Dagen 13-15 Dagen 16-22 Dagen 23-27 Dagen Dagelijks

*Als je* ***nooit diuretica hebt gebruik als middel om je lichaamsvorm/gewicht onder controle te houden,*** *ga dan verder met de volgende sectie.*

*Als je* ***ooit diuretica hebt gebruik als middel om je lichaamsvorm/gewicht onder controle te houden****, geef dan antwoord op de volgende vragen:*

**In de afgelopen TWEE weken, hoe groot was het deel van jou dat diuretica wilde gebruiken als middel om jouw lichaamsvorm/gewicht te beheersen?** (omcirkel een van de volgende percentages)

Een klein deel van mij Ongeveer de helft van mij Het grootste deel van mij

0% 10 20 30 40 50 60 70 80 90 100%

**In de afgelopen TWEE weken, hoe groot was het deel van jou dat bewust probeerde om het gebruik van diuretica te verminderen?** (omcirkel een van de volgende percentages)

Een klein deel van mij Ongeveer de helft van mij Het grootste deel van mij

0% 10 20 30 40 50 60 70 80 90 100%

**Stel dat je probeert om het gebruik van diuretica te verminderen, in hoeverre doe je dit dan voor jezelf (in tegenstelling tot voor anderen)?** (omcirkel een van de volgende percentages)

Nauwelijks voor mij Ongeveer half voor mij Vooral voor mij

0% 10 20 30 40 50 60 70 80 90 100%

**Stel dat je besluit om het gebruik van diuretica te verminderen, hoe zeker ben je dan dat je dat lukt?** (omcirkel een van de volgende percentages)

Helemaal niet zeker Heel zeker

0% 10 20 30 40 50 60 70 80 90 100%

**9. LICHAMELIJKE INSPANNING OM LICHAAMSVORMEN OF GEWICHT ONDER CONTROLE TE HOUDEN**

**Op hoeveel van de afgelopen 28 dagen heb jij intensieve lichamelijke inspanning verricht om je gewicht onder controle te houden, jouw lichaamsvorm of hoeveelheid vet te veranderen, of om calorieën te verbranden?** (Omcirkel een van de antwoordmogelijkheden)

Geen 1-5 Dagen 6-12 Dagen 13-15 Dagen 16-22 Dagen 23-27 Dagen Dagelijks

*Als je je* ***nooit lichamelijk hebt ingespannen om je lichaamsvorm/gewicht onder controle te houden,*** *ga dan verder met de volgende sectie.*

*Als je je* ***ooit lichamelijk hebt ingespannen om je lichaamsvorm/gewicht onder controle te houden****, geef dan antwoord op de volgende vragen:*

**In de afgelopen TWEE weken, hoe groot was het deel van jou dat zich intensief lichamelijk wilde inspannen?** (omcirkel een van de volgende percentages)

Een klein deel van mij Ongeveer de helft van mij Het grootste deel van mij

0% 10 20 30 40 50 60 70 80 90 100%

**In de afgelopen TWEE weken, hoe groot was het deel van jou dat bewust probeerde om de mate van het intensief lichamelijk inspannen te verminderen?** (omcirkel een van de volgende percentages)

Een klein deel van mij Ongeveer de helft van mij Het grootste deel van mij

0% 10 20 30 40 50 60 70 80 90 100%

**Stel dat je probeert om de mate van lichamelijke inspanning te verminderen, in hoeverre doe je dit dan voor jezelf (in tegenstelling tot voor anderen)?** (omcirkel een van de volgende percentages)

Nauwelijks voor mij Ongeveer half voor mij Vooral voor mij

0% 10 20 30 40 50 60 70 80 90 100%

**Stel dat je besluit om de mate van intensieve lichamelijke inspanning te verminderen, hoe zeker ben je dan dat je dat lukt?** (omcirkel een van de volgende percentages)

Helemaal niet zeker Heel zeker

0% 10 20 30 40 50 60 70 80 90 100%

**10. ANGST OM IN GEWICHT TOE TE NEMEN**

**Op hoeveel van de afgelopen 28 dagen ben jij bang geweest om in gewicht toe te nemen of om dik te worden?** (Omcirkel een van de antwoordmogelijkheden)

Geen 1-5 Dagen 6-12 Dagen 13-15 Dagen 16-22 Dagen 23-27 Dagen Dagelijks

*Als je je* ***nooit bang geweest om in gewicht toe te nemen,*** *ga dan verder met de volgende sectie.*

*Als je je* ***ooit bang bent geweest om in gewicht toe te nemen****, geef dan antwoord op de volgende vragen:*

**In de afgelopen TWEE weken, hoe groot was het deel van jou dat vast wilde houden aan jouw angst om in gewicht toe te nemen?** (omcirkel een van de volgende percentages)

Een klein deel van mij Ongeveer de helft van mij Het grootste deel van mij

0% 10 20 30 40 50 60 70 80 90 100%

**In de afgelopen TWEE weken, hoe groot was het deel van jou dat bewust probeerde om jouw angst voor gewichtstoename te verminderen?** (omcirkel een van de volgende percentages)

Een klein deel van mij Ongeveer de helft van mij Het grootste deel van mij

0% 10 20 30 40 50 60 70 80 90 100%

**Stel dat je probeert om jouw angst om in gewicht toe te nemen te verminderen, in hoeverre doe je dit dan voor jezelf (in tegenstelling tot voor anderen)?** (omcirkel een van de volgende percentages)

Nauwelijks voor mij Ongeveer half voor mij Vooral voor mij

0% 10 20 30 40 50 60 70 80 90 100%

**Stel dat je besluit om jouw angst om in gewicht toe te nemen te verminderen, hoe zeker ben je dan dat je dat lukt?** (omcirkel een van de volgende percentages)

Helemaal niet zeker Heel zeker

0% 10 20 30 40 50 60 70 80 90 100%

**11. ZICH DIK VOELEN**

**Op hoeveel van de afgelopen 28 dagen heb jij je dik gevoeld?** (Omcirkel een van de antwoordmogelijkheden)

Geen 1-5 Dagen 6-12 Dagen 13-15 Dagen 16-22 Dagen 23-27 Dagen Dagelijks

*Als je je* ***nooit dik hebt gevoeld,*** *ga dan verder met de volgende sectie.*

*Als je je* ***ooit dik hebt gevoeld****, geef dan antwoord op de volgende vragen:*

**In de afgelopen TWEE weken, hoe groot was het deel van jou dat vast wilde houden aan het gevoel van dik zijn?** (omcirkel een van de volgende percentages)

Een klein deel van mij Ongeveer de helft van mij Het grootste deel van mij

0% 10 20 30 40 50 60 70 80 90 100%

**In de afgelopen TWEE weken, hoe groot was het deel van jou dat bewust probeerde om je gevoel van dik zijn te verminderen?** (omcirkel een van de volgende percentages)

Een klein deel van mij Ongeveer de helft van mij Het grootste deel van mij

0% 10 20 30 40 50 60 70 80 90 100%

**Stel dat je probeert om jouw gevoel van dik zijn te verminderen, in hoeverre doe je dit dan voor jezelf (in tegenstelling tot voor anderen)?** (omcirkel een van de volgende percentages)

Nauwelijks voor mij Ongeveer half voor mij Vooral voor mij

0% 10 20 30 40 50 60 70 80 90 100%

**Stel dat je besluit om jouw gevoel van dik zijn te verminderen, hoe zeker ben je dan dat je dat lukt?** (omcirkel een van de volgende percentages)

Helemaal niet zeker Heel zeker

0% 10 20 30 40 50 60 70 80 90 100%

**12. BELANG VAN LICHAAMSVORM EN GEWICHT**

**Op hoeveel van de afgelopen 28 dagen heeft jouw gewicht en/of lichaamsvorm invloed gehad op hoe je je als persoon voelde (over jezelf dacht)?** (Omcirkel een van de antwoordmogelijkheden)

Geen 1-5 Dagen 6-12 Dagen 13-15 Dagen 16-22 Dagen 23-27 Dagen Dagelijks

*Als lichaamsvorm of gewicht* ***nooit van invloed is geweest op de manier waarop jij over jezelf denkt,*** *ga dan verder met de volgende sectie.*

*Als lichaamsvorm of gewicht* ***ooit van invloed is geweest op de manier waarop jij over jezelf denkt****, geef dan antwoord op de volgende vragen:*

**In de afgelopen TWEE weken, hoe groot was het deel van jou dat je lichaamsvorm of gewicht belangrijk vond?** (omcirkel een van de volgende percentages)

Een klein deel van mij Ongeveer de helft van mij Het grootste deel van mij

0% 10 20 30 40 50 60 70 80 90 100%

**In de afgelopen TWEE weken, hoe groot was het deel van jou dat bewust probeerde om je lichaamsvorm of gewicht minder belangrijk voor jezelf te maken?** (omcirkel een van de volgende percentages)

Een klein deel van mij Ongeveer de helft van mij Het grootste deel van mij

0% 10 20 30 40 50 60 70 80 90 100%

**Stel dat je probeert om lichaamsvorm of gewicht minder belangrijk voor jezelf te maken, in hoeverre doe je dit dan voor jezelf (in tegenstelling tot voor anderen)?** (omcirkel een van de volgende percentages)

Nauwelijks voor mij Ongeveer half voor mij Vooral voor mij

0% 10 20 30 40 50 60 70 80 90 100%

**Stel dat je besluit om lichaamsvorm en gewicht minder belangrijk voor jezelf te maken, hoe zeker ben je dan dat je dat lukt?** (omcirkel een van de volgende percentages)

Helemaal niet zeker Heel zeker

0% 10 20 30 40 50 60 70 80 90 100%

**APPENDIX 3**

RMQ validity and reliability

**TABLE 6.** RMQ scores per eating disorder symptom domain

|  | **Restriction** | | **Cognition** | | **Compensatory behavior** | | **Bingeing** | |
| --- | --- | --- | --- | --- | --- | --- | --- | --- |
|  | N | Mean (SD) | N | Mean (SD) | N | Mean (SD) | N | Mean (SD) |
| Precontemplation | 69 | 74.57 (21.55) | 76 | 62.26 (26.33) | 66 | 73.71 (24.38) | 7 | 45.71 (26.37) |
| Action | 69 | 20.51 (20.72) | 76 | 37.85 (24.13) | 66 | 32.10 (29.40) | 7 | 90.00 (8.16) |
| Internality | 69 | 27.61 (24.46) | 76 | 49.80 (32.17) | 64 | 37.47 (33.13) | 7 | 97.14 (4.88) |
| Confidence | 68 | 31.69 (25.64) | 76 | 18.18 (17.18) | 64 | 37.63 (29.91) | 7 | 52.86 (30.94) |

*Note:* Readiness and Motivation Questionnaire (RMQ) Precontemplation, Action, Internality, Confidence scores for each of the eating disorder symptom domains Restriction, Cognitions, Compensatory behaviors, Bingeing.

**TABLE 7.** Convergent validity

|  | **Pre-contemplation** | **Action** | **Internality** | **Confidence** | **EDI total** |
| --- | --- | --- | --- | --- | --- |
| Precontemplation | 1 |  |  |  |  |
| Action | -,32^**^ | 1 |  |  |  |
| Internality | -,43^**^ | ,61^**^ | 1 |  |  |
| Confidence | -,24^*^ | ,65^**^ | ,56^**^ | 1 |  |
| EDI total | ,58^**^ | -,26^*^ | -,44^**^ | -,25^*^ | 1 |

*Note:* Pearson correlations between Readiness and Motivation Questionnaire (RMQ) total precontemplation, action, internality, and confidence scores and the Eating Disorder Inverntory-3 (EDI-3) total score are reported. All correlations were in the expected direction. The EDI-3 is a self-report questionnaire that assesses the symptoms and psychological features of eating disorders, and has excellent reliability and adequate convergent and discriminant validity (Clausen et al., 2010). Participants respond on a 6-point Likert scale on 91 items, which yields composite scores on the following domains: eating disorder risk, ineffectiveness, interpersonal problems, affective problems, overcontrol and general psychological maladjustment. A higher score indicates that a psychological feature is more typical for the respondent as opposed to lower scores.

*** p* <.01, ** p* <.05. (1-tailed).

**TABLE 8.** Inter-item correlations RMQ

**8A.** Inter-item correlation matrix RMQ Precontemplation

|  | **1a** | **2.1a** | **2.2a** | **3a** | **4a** | **5a** | **6a** | **7a** | **9a** | **10a** | **11a** | **12a** |
| --- | --- | --- | --- | --- | --- | --- | --- | --- | --- | --- | --- | --- |
| 1a | 1.000 |  |  |  |  |  |  |  |  |  |  |  |
| 2.1a | .252 | 1.000 |  |  |  |  |  |  |  |  |  |  |
| 2.2a | .313 | -.624 | 1.000 |  |  |  |  |  |  |  |  |  |
| 3a | .039 | .282 | -.018 | 1.000 |  |  |  |  |  |  |  |  |
| 4a | .019 | -.042 | .169 | .296 | 1.000 |  |  |  |  |  |  |  |
| 5a | .124 | -.231 | .457 | -.083 | .392 | 1.000 |  |  |  |  |  |  |
| 6a | .321 | .304 | -.035 | .164 | .029 | .044 | 1.000 |  |  |  |  |  |
| 7a | .211 | -.082 | .283 | -.039 | .287 | .311 | .308 | 1.000 |  |  |  |  |
| 9a | .394 | .140 | .178 | .229 | -.095 | .013 | .148 | .107 | 1.000 |  |  |  |
| 10a | .428 | .420 | .090 | .349 | .129 | .039 | .199 | -.055 | .392 | 1.000 |  |  |
| 11a | .285 | .215 | .157 | .232 | .161 | .186 | .106 | -.037 | .389 | .662 | 1.000 |  |
| 12a | .620 | .256 | .230 | .160 | .093 | .164 | .177 | .117 | .497 | .604 | .448 | 1.000 |

**8B.** Inter-item correlation matrix RMQ Action

|  | **1b** | **2.1b** | **2.2b** | **3b** | **4b** | **5b** | **6b** | **7b** | **9b** | **10b** | **11b** | **12b** |
| --- | --- | --- | --- | --- | --- | --- | --- | --- | --- | --- | --- | --- |
| 1b | 1.000 |  |  |  |  |  |  |  |  |  |  |  |
| 2.1b | .318 | 1.000 |  |  |  |  |  |  |  |  |  |  |
| 2.2b | -.121 | -.267 | 1.000 |  |  |  |  |  |  |  |  |  |
| 3b | -.042 | -.058 | .052 | 1.000 |  |  |  |  |  |  |  |  |
| 4b | -.043 | .080 | .012 | .054 | 1.000 |  |  |  |  |  |  |  |
| 5b | -.016 | -.078 | -.008 | .163 | .474 | 1.000 |  |  |  |  |  |  |
| 6b | -.012 | .201 | -.060 | .082 | .182 | .185 | 1.000 |  |  |  |  |  |
| 7b | -.061 | .160 | .067 | .141 | .468 | -.048 | .420 | 1.000 |  |  |  |  |
| 9b | .109 | .512 | .128 | .319 | .081 | .049 | .092 | .162 | 1.000 |  |  |  |
| 10b | -.113 | .084 | .166 | .127 | .244 | .279 | .333 | .128 | .130 | 1.000 |  |  |
| 11b | -.046 | .189 | .004 | .168 | .104 | .003 | .209 | .144 | .218 | .473 | 1.000 |  |
| 12b | -.009 | .077 | .128 | .223 | .087 | -.074 | .184 | .263 | .245 | .392 | .323 | 1.000 |

**8C.** Inter-item correlation matrix RMQ Internality

|  | **1c** | **2.1c** | **2.2c** | **3c** | **4c** | **5c** | **6c** | **7c** | **9c** | **10c** | **11c** | **12c** |
| --- | --- | --- | --- | --- | --- | --- | --- | --- | --- | --- | --- | --- |
| 1c | 1.000 |  |  |  |  |  |  |  |  |  |  |  |
| 2.1c | .437 | 1.000 |  |  |  |  |  |  |  |  |  |  |
| 2.2c | -.085 | -.239 | 1.000 |  |  |  |  |  |  |  |  |  |
| 3c | -,.158 | -.056 | .221 | 1.000 |  |  |  |  |  |  |  |  |
| 4c | -.043 | .080 | .011 | -.007 | 1.000 |  |  |  |  |  |  |  |
| 5c | .012 | -.099 | .005 | -.071 | .609 | 1.000 |  |  |  |  |  |  |
| 6c | .166 | .140 | -.008 | .077 | .122 | -.039 | 1.000 |  |  |  |  |  |
| 7c | .124 | .086 | .099 | -.001 | .393 | .227 | .571 | 1.000 |  |  |  |  |
| 9c | .204 | .464 | .193 | .245 | .093 | .080 | -.028 | .078 | 1.000 |  |  |  |
| 10c | -.063 | .171 | .407 | .232 | .211 | .128 | .004 | .173 | .480 | 1.000 |  |  |
| 11c | .162 | .337 | .356 | .128 | .253 | .090 | .090 | .238 | .496 | .636 | 1.000 |  |
| 12c | .069 | .119 | .408 | .237 | .117 | .039 | .015 | .180 | .518 | .669 | .534 | 1.000 |

**8D**. Inter-item correlation matrix RMQ Confidence

|  | **1d** | **2.1d** | **2.2d** | **3d** | **4d** | **5d** | **6d** | **7d** | **9d** | **10d** | **11d** | **12d** |
| --- | --- | --- | --- | --- | --- | --- | --- | --- | --- | --- | --- | --- |
| 1d | 1.000 |  |  |  |  |  |  |  |  |  |  |  |
| 2.1d | .336 | 1.000 |  |  |  |  |  |  |  |  |  |  |
| 2.2d | .012 | -.304 | 1.000 |  |  |  |  |  |  |  |  |  |
| 3d | -.028 | -.168 | .533 | 1.000 |  |  |  |  |  |  |  |  |
| 4d | .099 | -.011 | -.011 | .086 | 1.000 |  |  |  |  |  |  |  |
| 5d | -.024 | -.121 | .021 | .331 | .661 | 1.000 |  |  |  |  |  |  |
| 6d | -.043 | .060 | -.012 | .095 | .222 | .072 | 1.000 |  |  |  |  |  |
| 7d | .036 | -.049 | .149 | .180 | .182 | .033 | .490 | 1.000 |  |  |  |  |
| 9d | .470 | .325 | .139 | .152 | .021 | .016 | -.086 | .095 | 1.000 |  |  |  |
| 10d | .143 | .197 | .238 | .055 | .152 | -.090 | .080 | .136 | .284 | 1.000 |  |  |
| 11d | .349 | .269 | .116 | .071 | .144 | .000 | .052 | -.003 | .087 | .503 | 1.000 |  |
| 12d | .253 | .192 | .079 | .038 | -.009 | -.168 | .137 | .093 | .268 | .703 | .454 | 1.000 |

*Note:* Corresponding questions of the Readiness and Motivation Questionnaire (RMQ) can be found in Appendix 2.
